# Listening in on difficult conversations: an observational, multi-center investigation of real-time conversations in medical oncology

**DOI:** 10.1186/1471-2407-13-455

**Published:** 2013-10-04

**Authors:** Brittany C Kimball, Katherine M James, Kathleen J Yost, Cara A Fernandez, Ashok Kumbamu, Aaron L Leppin, Marguerite E Robinson, Gail Geller, Debra L Roter, Susan M Larson, Heinz-Josef Lenz, Agustin A Garcia, Clarence H Braddock, Aminah Jatoi, María Luisa Zúñiga de Nuncio, Victor M Montori, Barbara A Koenig, Jon C Tilburt

**Affiliations:** 1 Biomedical Ethics Program, Mayo Clinic, Rochester, MN, USA; 2 Department of Health Sciences Research, Mayo Clinic, Rochester, MN, USA; 3 Knowledge and Evaluation Research Unit, Mayo Clinic, Rochester, MN, USA; 4 Johns Hopkins Berman Institute of Bioethics, Baltimore, MD, USA; 5 Johns Hopkins School of Medicine, Baltimore, MD, USA; 6 Johns Hopkins Bloomberg School of Public Health, Baltimore, MD, USA; 7 Department of Medicine, University of Southern California Norris Comprehensive Cancer Center, Los Angeles, CA, USA; 8 Division of General Internal Medicine, Stanford University, Stanford, CA, USA; 9 Division of Medical Oncology, Department of Oncology, Mayo Clinic, Rochester, MN, USA; 10 Division of Global Public Health, University of California San Diego, La Jolla, CA, USA; 11 Institute for Health and Aging, University of California San Francisco, San Francisco, CA, USA; 12 Division of General Internal Medicine, Mayo Clinic, Rochester, MN, USA

**Keywords:** Cancer, Oncology, Physician-patient communication

## Abstract

**Background:**

The quality of communication in medical care has been shown to influence health outcomes. Cancer patients, a highly diverse population, communicate with their clinical care team in diverse ways over the course of their care trajectory. Whether that communication happens and how effective it is may relate to a variety of factors including the type of cancer and the patient’s position on the cancer care continuum. Yet, many of the routine needs of cancer patients after initial cancer treatment are often not addressed adequately. Our goal is to identify areas of strength and areas for improvement in cancer communication by investigating real-time cancer consultations in a cross section of patient-clinician interactions at diverse study sites.

**Methods/design:**

In this paper we describe the rationale and approach for an ongoing observational study involving three institutions that will utilize quantitative and qualitative methods and employ a short-term longitudinal, prospective follow-up component to investigate decision-making, key topics, and clinician-patient-companion communication dynamics in clinical oncology.

**Discussion:**

Through a comprehensive, real-time approach, we hope to provide the fundamental groundwork from which to promote improved patient-centered communication in cancer care.

## Background

“You have cancer.” Approximately 1.6 million people in the United States heard these frightening words in 2012 in the context of a new cancer diagnosis [1]. In delivering a diagnosis, making decisions about life-altering treatment, and ultimately helping patients navigate through diagnosis, treatment, survivorship, and/or end-of-life care, oncology clinicians carry a deep responsibility to offer information and support in a manner that will be most helpful to their patients – a task which must be individualized for each interaction. Clinicians serve as technical experts, while patients hold expert knowledge about their own feelings, life circumstances and preferences; both play a crucial role informing in treatment decisions. Family and friends can also play an important contributing role in the process of diagnostic and treatment decision-making and in offering support in the midst of and after treatment. Clinicians can help facilitate this multi-faceted conversation through patient-centered, empathic interactions – arguably in a manner consistent with a shared-decision making model [2].

As a fundamental component of quality health care, patient-centered communication is an important area for investigation in cancer care. Previous studies have shown that patient-centered communication can improve the patient experience, patient health status and outcomes, and the efficiency of medical care [3-6]. Furthermore, other studies indicate that poor communication in cancer care can result in economic, social, psychological, emotional, and collateral costs to patients, their support networks, clinicians, the cancer care system, and society more widely [6].

Due to the gravity of the diagnosis, communication between cancer patients and their treating clinicians may be emotionally intense; patient needs likely vary depending on tumor type, age, sex, health literacy, social and cultural norms and where a patient is located along the cancer care continuum. The 2006 Institute of Medicine report, *From Cancer Patient to Cancer Survivor*: *Lost in Transition*, concluded that many of the routine needs of cancer patients after initial cancer treatment were not being adequately addressed [7]. The topics that are often of most importance to patients include quality of life, sexual dysfunction, the safety of complementary and alternative medicines (CAM) and other important questions which may or may not be routinely addressed in consultation. Discussing uncertainty, risk and care options also pose challenges to patient-clinician communication [8]. This disconnect in communication has been documented among Latinos living with HIV and their clinicians [9,10], however, there is a significant research gap in underlying factors that influence cancer patient-clinician communication, especially in ethnic minority cancer patients. Further, the role of friends and family in cancer conversations remains an important but under-studied element of cancer patient care [11]. Improved understanding of the patient-clinician-companion dynamic could help identify existing strengths and areas for improvement in this domain and lead to improved patient adherence to therapy and clinical care visits [12].

Without a detailed assessment of the challenges and opportunities for achieving a more patient-centered dynamic in existing clinical consultations, improving clinician-patient interactions in cancer care could be difficult, haphazard, and unsustainable. A detailed description of cancer decision-making processes surrounding key topics important to patients, but that fall outside the scope of cancer therapeutics could enable feasible, sustainable practice-based interventions to be identified, tested, and implemented. Developing a comprehensive picture of what patient-clinician-companion dynamics in cancer care look like is the first step in improving the quality of these interactions.

The existing literature assessing the measurement of patient-centered communication in cancer care suggests that the communication process can be divided into six key domains: *exchanging information*, *fostering healing relationships*, *managing uncertainty*, *recognizing and responding to emotions*, *making decisions*, *enabling self*-*management* and *patient navigation*, as well as cross-cutting themes among these [11]. We build on insights from this growing body of work in an ongoing observational study designed to fill in gaps in the existing data on patient-clinician-companion communication in cancer care by focusing on features of real-time clinical discussions as they occur in practice. Below we describe a study in which we involve multiple institutions, utilize mixed empirical methods, and employ a short-term longitudinal, prospective follow-up component to begin assessing what really goes on in oncology care discussions across a diversity of patient populations and a variety of tumor types and practice settings. Our approach is interdisciplinary, drawing upon existing conceptual frameworks of communication in cancer and addressing questions with qualitatively and quantitatively tools. This paper describes the current state of patient-clinician cancer communication and identifies specific gaps that ongoing research must address. Through our comprehensive, real-time approach, we hope to provide a foundation upon which to develop methods for enhancing patient-centered communication in cancer care.

### Hypothesis and rationale

We hypothesize that clinician-patient conversations about key topics such as quality of life, cost, sexual function, or complementary and alternative therapies will lack important elements of informed decision-making compared to conversations focused on cancer treatment options and symptom management. We further hypothesize that the degree of patient-centeredness in cancer consultations (using standardized metrics) will be an important predictor of discussion content and how these topics are discussed. Our rationale for this research is that eventual interventions to promote patient-centered communication in cancer must start with a detailed characterization of actual discussions between cancer patients and their clinicians within a broad cross-section of oncology care. To date such studies have only addressed therapeutic treatment decision making in breast cancer [13] and end of life decision making [14].

### Objectives

The overall objective of this line of research is to improve the patient experience in the communication process by first characterizing existing care conversations in a variety of clinical settings in medical oncology. The specific aims of the research are outlined in Table 1.

**Table 1:** Study aims

	
**Aim 1.**	**To richly characterize the dynamics and quality of patient-clinician-companion interactions in routine cancer care consultations by documenting the frequency, duration, and content of conversations about key issues that are important to patients in their care.**
**1a.**	To describe the frequency, duration and content of routine cancer consultations surrounding key challenging topics in the clinical dialogue.
**1b.**	To examine in-depth the fundamental psycho-social dynamics of deliberations that occur between patients and clinicians during routine cancer care consultations.
**1c.**	To assess the comprehensiveness of these discussions pertaining to key elements of informed decision-making.
**1d.**	To assess the degree of content concordance between topics raised in the recorded conversations and what is documented in the medical record for each of the key topics.
**Aim 2.**	**To identify key characteristics of cancer consultation participants and dialogue that influence subsequent clinical actions and short term outcomes.**
**2a.**	To identify clinician characteristics associated with the discussion of topics in the key topic list raised during a routine cancer consultation.
**2b.**	To identify patient clinical, demographic, and psychosocial characteristics associated with the discussion of topics in the key topic list raised during a routine cancer consultation.
**2c.**	To determine if the patient-centeredness of patient-clinician dialogue predicts which topic areas are discussed and the subsequent decisions that are made in a patient’s care across English and Spanish-speaking (and mixed) care contexts.

### Methods/design

**Figure 1:**
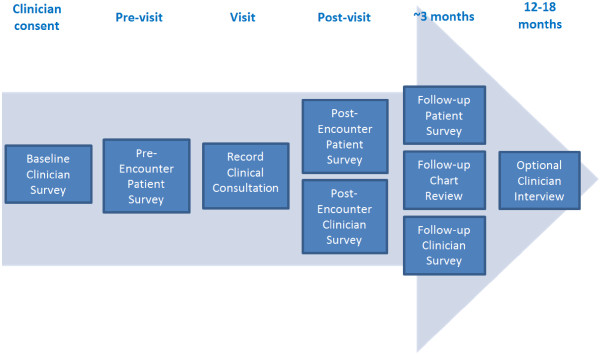
Schematic of data collection modes & timeline.

This study has been approved by the Mayo Clinic Institutional Review Board. The overall design of this study is prospective and observational. We will use real-time observation of clinical interactions as the main means of data collection augmented by self-reported survey measures and medical record review (Figure 1). This prospective design will allow us to capture a clear picture of what is really happening in routine clinical interactions with minimal intrusion.

Our data collection approach seeks to discover inductively the characteristics of high quality communication while examining discussions for known features of high quality communication. We will extend this mentality into the analysis phase described below by using a combination of emergent and *a priori* coding techniques in which we allow new themes to emerge while identifying specific issues and dynamics related to our foundational assumptions and specific aims. Qualitative methods will allow us to examine the features of conversations in this context without restricting that analysis to a single analytic viewpoint. Without being constrained by a particular conceptual model, we will employ a variety of well tested, broadly accepted analytic techniques in order to characterize real-time interactions between oncology clinicians and their patients. In applying an approach that is both qualitative and quantitative, we hope to build a comprehensive picture of clinical interactions in oncology with sufficient depth to inform future efforts to improve the quality of these deliberations.

### Participants & recruitment

#### Clinicians

In order to obtain patient/clinician dyads engaged in clinical conversation, we will begin by recruiting clinicians. We will enroll medical oncology clinicians, including physicians, nurse practitioners, physician assistants, and senior fellows in medical oncology who actively practice with at least 20% clinical time. Clinicians will be recruited from three hospitals: Mayo Clinic-Rochester, University of Southern California-Norris Comprehensive Cancer Center, and Los Angeles County Hospital.

We aim to accrue a total of 8–12 patients per enrolled clinician. Eligible patients must be age 18 years or older, speak English or Spanish, must not be enrolled in hospice, and have received histological confirmation of any solid tumor malignancy including brain, breast, endocrine, gastrointestinal, genitourinary, gynecological, head/neck, lung, melanoma, or sarcoma malignancy at all points of the cancer continuum which we define as initial diagnosis, early initial treatment, mid-initial treatment, post-treatment/survivorship/remission, recurrence & undergoing treatment, and end-stage disease. We intentionally developed broad inclusion criteria within the more restricted chronic disease category of cancer so as to keep the study focused on an important population. In total, we expect to include 60 medical oncology clinicians (45 faculty-level medical oncologists, approximately 10–15 oncology nurse practitioners, and 6–8 senior hematology/oncology fellows) and 600 medical oncology patients across the three sites.

After compiling lists of eligible clinicians at each study site, we will invite these individuals to participate via phone, email, or in-person interactions. Written informed consent will be obtained from clinicians who volunteer to participate. We decided on written informed consent as our standard operation for several reasons. From a human subjects protection perspective, if our IRB protocol at one study site permitted only verbal consent but the other two study sites require written consent, we did not want to jeopardize having to revise the overall study protocol to accommodate potential concerns that could be raised on the secondary study sites. Thus, although this would be a legitimate circumstance in which to utilize verbal consent, we opted for the more conservative written consent. Moreover, given the potential sensitivity of topics discussed, and the general familiarity that oncology clinicians have with written consent, we thought they would consider written consent a more standard and robust approach.

#### Patients

Prior to approaching patients for consent during an agreed upon half-day with clinicians, study personnel will review with clinicians a list of the day’s eligible patients to give clinicians an opportunity to decline studying interactions with a particular patient for whom the interaction would be too sensitive. Approved patients will then be approached in their exam room or in another private room in the order that they will be seen by their clinician. If the patient expresses interest in participating after a brief introduction to the study, study personnel will undertake a full written consent process (and oral consent for any companions who are present). In this process the study coordinator will walk through the risks and benefits of the proposed study, allowing ample time for discussion and clarification. To ensure voluntariness, they will re-iterate that the patient’s care will change in no way. Consented patients will be offered a 4-hour parking voucher as a small token of thanks.

### Data collection

Our main modes of data collection in this study will include patient and clinician surveys, an audio-recorded clinical conversation, medical record review, and optional interviews with clinicians. Survey instruments for this study were developed and adapted from a variety of validated measures of patient reported outcomes, quality of life, satisfaction, and health behaviors from existing widely used tools whenever possible [15-18]. Specific time points of quantitative data collection can be found in Table 2.

**Table 2:** Table of quantitative variables and time points of collection

	**Clinician consent**	**Pre-visit**	**Visit**	**Post-visit**	**~3 months**
***Baseline Clinician Survey***					
Demographics	✓				
Professional practice	✓				
***Baseline Patient Survey***					
Quality of life					
*Overall*		✓		✓	
*Emotional well*-*being*		✓		✓	
*Physical well*-*being*		✓		✓	
*Intellectual well*-*being*		✓		✓	
*Social activity*		✓		✓	
*Spiritual well*-*being*		✓		✓	
*Pain*		✓		✓	
*Fatigue*		✓		✓	
*Support from friends*/*family*		✓		✓	
*Treatment burden on self*		✓		✓	
*Treatment burden on family*		✓		✓	
Functional literacy		✓		✓	
Demographics		✓		✓	
***Observation***					
RIAS coding			✓		
***Post-Encounter Patient Survey***					
Agenda setting in visit				✓	
Patient-centeredness				✓	
Concerns not discussed				✓	
Shared decision-making				✓	
Clinician rating				✓	
Visit satisfaction				✓	
Quality control- observation bias				✓	
***Post-Encounter Clinician Survey***					
Patient position on cancer control spectrum				✓	
Decision made				✓	
Quality of visit				✓	
***3-Month Follow up Patient Survey***					
Quality of life		✓			✓
*Overall*		✓			✓
*Emotional well-being*		✓			✓
*Physical well-being*		✓			✓
*Intellectual well-being*		✓			✓
*Social activity*		✓			✓
*Spiritual well-being*		✓			✓
*Pain*		✓			✓
*Fatigue*		✓			✓
*Support*		✓			✓
*Treatment burden on self*		✓			✓
*Treatment burden on family*		✓			✓
Cancer care decision-making preference and experience					✓
CAM use					✓
***Chart Review***					
Location of patient care					✓
Insurance					✓
Family cancer history					✓
General cancer information					✓
Complementary and integrative medicine referrals					✓
Cancer related CAM use					✓
***Post-Study Clinician Survey***					
Discussing CAM					✓
Discussing psychosocial issues					✓
Discussing End-of-life care					✓

#### Initial clinician and patient surveys

Once a clinician consents to participate in the study, study staff will administer a baseline survey that will collect basic demographic and professional practice characteristics of clinicians including age, sex, race/ethnicity, number of years in practice, and any general training in communication. Study personnel will also administer a survey (hereafter referred to as the “pre-encounter patient survey”) to each patient immediately following the informed consent process and immediately prior to their oncology appointment. The pre-encounter patient survey will assess patient demographics, health literacy [19,20], and quality of life [21].

#### Audio recorded clinical conversation

For the second part of data collection, study staff will place a handheld digital audio recorder in the enrolled patient’s exam room and turn it on at the start of the patient-clinician encounter. Patients and clinicians will have the option of turning the recorder off at any time and will be trained on how to do this. A red light on the recorder, which signals that it is recording, will ensure that the clinician and patient know at all times whether the recorder is on or off. At the end of the visit, the recorder will be turned off and the recordings immediately transferred and saved to an internal server accessible only to our research team.

We will use an online editing tool (Audacity™) to remove any personal identifiers from the recordings before they are transcribed and sent to our collaborating analysis site. The files will be uploaded to a password-protected flash drive and mailed to our analysis site for analysis. During this process we will “flag” regions on the recording where the key topics are discussed. These “flags” will anchor subsequent topical qualitative and quantitative analyses.

#### Post-encounter patient and clinician surveys

Immediately following the clinical consultation, patients will be asked to fill out a second survey. This “post-encounter patient survey” will collect information about the patient’s perspective on the just-concluded visit. The post-encounter patient survey was developed using preexisting measures including the CAHPS Clinician & Group Surveys - Visit Survey 2.0 (https://cahps.ahrq.gov/clinician_group/) (modified to instruct patients to answer about the encounter that just occurred with their cancer clinician), and the SDM Q-9 [18]. In this survey, patients assess patient and clinician roles in the conversation, the extent to which communication with their clinician was patient-centered, if a specific decision was made, as well as report about the degree of shared decision-making present in that deliberation using the above metrics. It will also document if a patient feels that any of his or her important concerns were not discussed in the visit as well as whether any key topics, including CAM, symptom management, and emotional or social concerns were discussed. More global questions about the visit include a clinician rating and an overall score of the patient’s satisfaction with the visit. A final question serves as a quality control measure, asking how comfortable the patient felt being recorded to determine if the presence of the recorder in the conversation may have influenced the dynamic.

Clinicians will also be asked to complete a second survey immediately following their appointment with a study patient. The one-page “post-encounter clinician survey” will address patient and visit-specific topics from a clinician perspective including the patient’s location on the cancer care continuum (i.e. initial diagnosis, early initial treatment, mid-initial treatment, post-treatment/survivorship/remission, recurrence & undergoing treatment, and end-stage disease) and the clinician’s perception of the quality and effectiveness of the encounter (i.e. “I felt that my time with this patient today was well spent”; “I established rapport with this patient today”; “I was able to obtain an accurate and detailed medical history from this patient”; “I think this patient requires a lot of emotional support”; “I think that this patient is coping well with his/her cancer treatment and side effects”; “Overall I was satisfied with this encounter today”). In addition, this survey will ask clinicians if they felt a specific decision about the patient’s care was made during the visit, enabling us to subsequently assess the degree of concordance with patient-self ratings of the same measure and concordance with chart review. Although there is significant debate about whether discrete “decisions” reflect the complex lived experiences of patients [22], being able to document concordance and discordance in these ratings as well as complementing these quantitative variables with more inductive, qualitative methods should further elucidate those debates.

#### Follow-up patient survey and chart review

Three months following direct-observation recording, we will mail each study patient a paper follow-up survey including a cover letter and an addressed, stamped return envelope. This survey will allow us to longitudinally assess any changes in quality of life. Additionally, it will help us assess our list of key topics as well as patient decision-making preferences. Having these measures at the end of the study period limits the effects of observer bias on patient and provider behavior.

At the same time follow-up surveys are being mailed, study staff will conduct medical record reviews for each study patient. Assessment of each patient’s chart will permit us to examine major medical events as well as any documentation of key topics in clinical notes.

#### Follow-up clinician survey and optional interview

After each enrolled clinician has reached his or her maximum number of study patient interactions (i.e. 8–12), a follow-up clinician survey will be administered. Because clinicians may differ with respect to their comfort level discussing potentially sensitive topics with their patients, the follow-up survey will assess the attitudes and behaviors of clinicians with regard to discussing these topics with their patients at a point in the study where our questions do not influence their clinical behavior observed. Specifically, this survey will ask about discussing complementary and alternative medicine use, psychosocial issues, and end-of-life care.

Enrolled clinicians will also be asked if they are interested in participating in an optional semi-structured interview at the conclusion of the study. The interviews will provide an opportunity to debrief clinicians on the aims of the study as well as a chance to delve into their views on shared decision-making, communication surrounding key topics, and challenges and opportunities for communication in a medical oncology setting. Using this approach will allow us to discuss previously unknown concerns that can only emerge through inductive approaches. For instance clinicians talking about CAM as part of a larger process of helping patient reconcile their healing experience with the recommendations of an indigenous healer from their home village or discussing the role of relatively benign “immune boosting” supplements in order to encourage the patient to complete chemo therapy.

*Please note: complete data collection instruments are accessible in Additional file 1. More detailed standard operating procedures are available upon request.

### Study Pre-test

In an effort to elucidate and address potential methodological or logistic challenges prior to actual study implementation, we conducted a study pre-test approximately three months before the start of participant enrollment and data collection. All clinician and patient recruitment, enrollment, and data collection procedures (including audio-recording of appointments and dissemination of surveys) were pre-tested with three oncology clinicians and 15 patients at Mayo Clinic. This pre-test process proved invaluable in helping us identify and address procedural issues such as the location and timing of patient enrollment, questions or confusion about survey items needing re-wording, as well as simply to establish rapport and a good working relationship with the clinical desk staff in medical oncology.

### Analysis

All clinician and patient survey responses will be collected, double-entered, and managed by study staff using REDCap electronic data capture tools hosted at Mayo Clinic [23]. REDCap (Research Electronic Data Capture) is a secure, web-based application designed to support data capture for research studies, providing: 1) an intuitive interface for validated data entry; 2) audit trails for tracking data manipulation and export procedures; 3) automated export procedures for seamless data downloads to common statistical packages; and 4) procedures for importing data from external sources.

After data collection and entry we will explore what the data mean for each study aim (see Table 1):

#### Aim 1

To characterize the content of the recorded conversations, we will employ the Roter Interaction Analysis System (RIAS), one of the most widely used and extensively validated approaches to quantitative discourse analysis of medical encounters [24,25]. Coders blinded to the study’s hypotheses will use RIAS to categorize each utterance of the clinical encounter into 40 categories. With this system’s flexibility, codes can be individually applied to a piece of the interaction or combined with one another to summarize dialogue. These categories will organize the data, providing a foundation upon which we can begin to assess the dynamics of these conversations. Through this method we will examine the data quantitatively, assessing dialogue through the four-function Communication Model which informs RIAS [26]. This validated method has been applied in a variety of medical settings, including oncology [26,27].

In addition to quantitative techniques for analyzing the recorded conversations, we will use qualitative content analysis to characterize the fundamental nature of discussions about key topics. In our data analysis we will use a combination of *a priori* and emergent coding techniques that will allow us to search for key topics of interest, while exploring the questions, “What is this about?” and “What is being referenced here?” in a manner that will allow new themes to emerge [28,29]. A priori techniques look for pre-defined categories like “expressions of empathy” or other known important psychosocial categories. Emergent techniques will maintain a posture of receptivity to elements of meaning that may not have been pre-specified. For instance, even if our theoretical models do not specify it, we might in the context of analysis, discover that “tone of voice” or “sharing of personal anecdotes” shape how dialogue is shaped. Used widely in ethnographic and direct observational data analysis, this approach to conversation analysis contextualizes participants’ understanding, makes comparisons, and tracks variations in meaning across specific cases [30-33].

After coding with RIAS, during which we will identify instances of key topics brought up during the discussion, we will carefully dissect the content of those discussions using a combination of two existing measures of decision quality: the OPTION scale and the IDM-18. The IDM-18 is a validated measure of key elements of informed decision-making, while the OPTION scale rates the degree to which patients were engaged in decision-making about their care [34,35]. Study team members will apply these measures to flagged recordings and each will rate interactions with an approach similar to video analyses we have done in the past [36]. We will ensure a high degree of inter-rater reliability on a subset of recordings before applying the full scoring systems to the entire data set. Both of these analysis techniques are subject to their own strengths and weaknesses [37]. However, when used in tandem, we believe that they will begin to sketch a more comprehensive picture of decision-making quality in this context.

A follow-up medical record review three months after the audio-recorded clinical encounter will allow us to review patient participants’ medical records for study-related information. This review will have the capacity to assess documentation of any actions related to the key topics starting initially with complementary and alternative therapies. Using these records, we will apply accepted methods of medical record chart review [38] and document all major events in the patient participants’ treatment course to date as well as determine whether aspects related to the key topics mentioned above were documented. Medical record review data will be double-entered using a REDCap database.

#### Aim 2

To determine how different characteristics of cancer consultation participants and their dialogue influence the discussions and subsequent clinical actions, we will conduct univariate and multivariate statistical analyses. Information for these analyses will be obtained from the codes assigned to the audio recordings and information from the surveys and chart abstraction. We will employ Pearson chi-square and/or Fisher’s exact tests (for univariate testing) followed by multivariate logistic regression models to identify clinician and patient clinical, demographic, as well as psychosocial characteristics associated with having discussed key topics.

### Potential limitations

In a large mixed-method, multi-site study like ours, we may face a variety of problems that could impede our progress. We may face difficulties in the rate of accrual, participant (patient and/or clinician) discomfort with being recorded, the Hawthorne effect, concerns related to multi-lingual data collection and analysis, social-desirability or premature disclosure of study hypotheses. Each potential challenge will be addressed as follows.

As currently conceived, we envision recruiting 20–40 patient participants per month for approximately 30 months. If we encounter challenges with the rate of accrual, we have the capacity to extend our data collection an extra year into the study’s final year while simultaneously undertaking all necessary analyses. Participant anxiety about being recorded can be addressed by reiterating that patients and clinicians may turn the recording device off at any time during the appointment.

Regarding the Hawthorne effect, although there are methods for ensuring that observed behavior is truly naturalistic, our experiences recording decision-aid trials at Mayo Clinic as well as other studies using direct observation have shown that patients and clinicians adapt to being recorded very quickly, soon ignoring the presence of recording devices. While it is true that recorded visits may capture "best behavior," it is unlikely that this is systematically interpreted by physicians in a way that would jeopardize the validity of findings. The issue of performance bias in response to tape recording has been addressed in several studies [39-42]. All have found that the effect is minimal. Included among these is a study in which the content of video recordings of physicians who were and were not informed that recordings were being made found no statistically significant differences in length of visit or in the number or nature of the problems discussed [41].

Our analysis may be complicated by multi-lingual data collection and analysis. The complications related to translation, back translation, and validity of study instruments used in multiple languages are well known [43-45]. We will mitigate these problems by using optimal data handling practices for translation and back translation, using previously validated Spanish-language versions of all study tools whenever possible, and utilizing the expertise of a team member conversant with Latino cultures in Southern California as well as using Spanish-speaking analytic expertise for our RIAS coding.

In order to prevent participation bias, we intend not to disclose the aims of our study to research participants throughout the duration of data collection. This could be very important among clinicians. We will assess this qualitatively in the interviews and quantitatively in the follow up survey. Although we cannot anticipate all of the challenges we might face, the vast experience of the study team in accruing participants for research studies and in recording real-time decision-making processes in clinical consultations has prepared us to resolve issues as they arise.

It is also possible that our detailed and in-depth consent procedures may in some way bias or prime patients for a different kind of conversation than they might have otherwise had. In order to satisfy regulatory stakeholders and conduct the study with integrity, we must accept the limits this possibility this may bring.

## Discussion

We have described an ongoing large multi-center observational study designed to investigate and characterize clinical interactions between clinicians and patients as they occur in oncology as well as identify key factors that influence decision-making about important topics in cancer care. Presenting these methods here will allow for other authors to build upon and critique our approach while data are still being generated. In attempting to capture a picture of any aspect of health care, inherent difficulties may arise in balancing the breadth and depth of the of inclusion criteria for a heterogeneous clinical population. One of the challenges in implementing a study like this involves determining the most useful sample group. We intentionally developed broad inclusion criteria within the more restricted chronic disease category of cancer so as to keep the study focused on an important population.

Having large, but manageable, groups of clinicians is the single most important feature in determining whether a study can capture a breadth of variability in communication behavior, as it is typically clinicians, not necessarily patients, who contribute the greatest variability in communication behavior [46]. However, our existing study sites, although diverse, are not nationally representative. Mayo Clinic’s large oncology practice makes it an ideal site for completing a study of this magnitude. Although including more sites may have been preferable, we were concerned that a large number of sites would severely decrease the study’s feasibility. We anticipate that the patient sample accrued at USC Norris and LA County Hospital will be more heterogeneous than at Mayo Clinic and, specifically at LA County, will include a large proportion of un- or under-insured minority patients.

This study provides an important opportunity to assess both the difficulties and opportunities for improving the quality of discussions in a cancer care setting and will thereby yield an invaluable baseline description for future interventional studies. Our study will explore the nature of these interactions in order to pinpoint the strengths and weaknesses of these deliberations as they exist today. In doing this, we hope to inform future interventions for improving the quality of discussions in the cancer care context.

## Competing interests

The authors declare that they have no competing interests.

## Authors’ contributions

KJY, AK, MRE, GG, DLR, SML, HJL, AAG, CHB, AJ, MLZ, VMM, BAK, and JCT were involved in the design of the study. BCK, KMJ, CAF, ALL, and JCT drafted the manuscript. The manuscript has been read and approved by all authors.

## Pre-publication history

The pre-publication history for this paper can be accessed here:

http://www.biomedcentral.com/1471-2407/13/455/prepub

## Supplementary Material

Additional file 1 Data collection instruments. Click here for file
