# Supplementary material for: Listening in on difficult conversations: an observational, multi-center investigation of real-time conversations in medical oncology
Source: BMC Cancer. 2013 Oct 4;13:455. doi: 10.1186/1471-2407-13-455 (PMC3850997; doi:10.1186/1471-2407-13-455)
Supplement: Additional file 1 — Data collection instruments. [file 1471-2407-13-455-S1.pdf]

BASELINE SURVEY FOR CLINICIANS

Thank you for participating in our study on the quality of conversations between cancer patients and their clinicians. We hope you will take 2 minutes to complete this brief survey.

SECTION A – About You

1. What is your sex?

☐1 Male
☐2 Female

2. What is your current age?

_____ years

3. Do you consider yourself Hispanic or Latino?

☐1 Yes
☐2 No

4. How do you classify your race?

☐1 Asian
☐2 Black or African-American
☐3 American Indian or Alaskan Native
☐4 White or Caucasian
☐5 Other ________________________________

- Please proceed to page 2 -

SECTION B – Your Professional Practice

5. What year did you graduate from medical school (if MD) or receive your highest level of professional training (if NP or PA)?

__ __ __ __

6. On which of the following organ type(s) does your practice focus?
(mark up to 3)

☐1 Brain
☐2 Breast
☐3 Endocrine
☐4 Gastrointestinal
☐5 Genitourinary
☐6 Gynecological
☐7 Head/neck
☐8 Lung
☐9 Melanoma
☐10 Sarcoma
☐11 Other: ____

7. Have you ever had any formal training/coursework in cancer communication (e.g. Oncotalk)?

☐1 Yes
☐2 No

8. At the end of this study, we would like to conduct brief in-person interviews to learn more about patient-clinician communication in your clinic. Would you be willing to be re-contacted to consider participating in an interview?

☐1 Yes, I am willing to be re-contacted.
☐2 No thanks.

Thank you!

Please return your completed survey to study coordinator, Cara Fernandez (Internal mailing address: Plummer 3 CTSA)

CANCER PATIENT EXPERIENCE AND COMMUNICATION STUDY

PRE-ENCOUNTER PATIENT SURVEY

MAYO CLINIC

Thank you for participating in our study on communication between cancer patients and their providers.

Please take a few minutes now, before your appointment, to complete this brief survey.

When you are finished, please return the survey to the study coordinator, Cara Fernandez, or the main desk where you checked in for your appointment.

If you have any questions about this study, please contact:
Cara Fernandez, study coordinator: 507-266-1897
Katie James, project coordinator: 507-293-0707

PRE-ENCOUNTER PATIENT SURVEY

SECTION A – Your Quality of Life

1. Please circle the number (0-10) best reflecting your response to the following questions that describe your feelings during the past week, including today. How would you describe:

a. your overall quality of life?

0 1 2 3 4 5 6 7 8 9 10
As bad as it can be — As good as it can be

b. your overall emotional well-being?

0 1 2 3 4 5 6 7 8 9 10
As bad as it can be — As good as it can be

c. your overall physical well-being?

0 1 2 3 4 5 6 7 8 9 10
As bad as it can be — As good as it can be

d. your overall mental (intellectual) well-being?

0 1 2 3 4 5 6 7 8 9 10
As bad as it can be — As good as it can be

e. your level of social activity?

0 1 2 3 4 5 6 7 8 9 10
As bad as it can be — As good as it can be

f. your overall spiritual well-being?

0 1 2 3 4 5 6 7 8 9 10
As bad as it can be — As good as it can be

g. the frequency of your pain?

0 1 2 3 4 5 6 7 8 9 10
No pain — Constant pain

h. the severity of your pain, on the average?

0 1 2 3 4 5 6 7 8 9 10
No pain — Pain as bad as you can imagine

i. your level of fatigue, on the average?

0 1 2 3 4 5 6 7 8 9 10
No fatigue — Constant tiredness

j. your level of support from friends and family?

0 1 2 3 4 5 6 7 8 9 10
No support — Highest level of support

2. What has been the effect of your cancer diagnosis and treatment on your overall quality of life? (Please circle the appropriate number)

0	1	2	3	4	5	6	7	8	9	10
Totally ruined quality of life										No effect on quality of life

Comments:____

3. What has been the effect of your cancer diagnosis and treatment on your family's quality of life? (Please circle the appropriate number)

0	1	2	3	4	5	6	7	8	9	10
Totally ruined quality of life										No effect on quality of life

Comments:____

SECTION B – Seeking and Understanding Health Information

Please answer the following questions about how you seek and understand health information, checking only one box for each item:

	None of the time	A little of the time	Some of the time	Most of the time	All of the time
**4. How often do you have someone help you read hospital materials?**	☐1	☐2	☐3	☐4	☐5
**5. How often do you have problems learning about your medical condition because of difficulty understanding written information?**	☐1	☐2	☐3	☐4	☐5
**6. How often do you have trouble understanding medical information spoken to you by doctors and nurses?**	☐1	☐2	☐3	☐4	☐5

7. How confident are you filling out medical forms by yourself?

☐1 Not at all confident
☐2 A little confident
☐3 Somewhat confident
☐4 Very confident
☐5 Extremely confident

SECTION C – About You

8. Where were you born?

☐1 United States ☐2 Mexico ☐3 Other country: _______________

9. Do you consider yourself Hispanic or Latino?

☐1 Yes ☐2 No

10. How do you classify your race? (Check only one)

☐1 Asian
☐2 Black or African-American
☐3 American Indian or Alaskan Native
☐4 White or Caucasian
☐5 Other ____

11. In what language(s) do you speak?

☐1 English only
☐2 Mostly English, some Spanish
☐3 Spanish and English, about equally
☐4 Mostly Spanish, some English
☐5 Spanish only
☐6 Other language: _______________________________________

12. What is the highest grade or year of school you completed?

☐1 Less than high school
☐2 Grade 12 or GED (high school graduate)
☐3 College 1 year to 3 years (some college or technical school)
☐4 College graduate
☐5 Post-graduate study
☐6 Other:____

13. What is your annual household income from all sources?

☐1 Less than $10,000
☐2 $10,000 to less than $20,000
☐3 $20,000 to less than $35,000
☐4 $35,000 to less than $50,000
☐5 $50,000 to less than $75,000
☐6 More than $75,000

THANK YOU!
Please return your completed survey to Cara Fernandez, study coordinator.
Following your appointment with your cancer provider today, we will ask you to complete one more short survey.

CANCER PATIENT EXPERIENCE AND COMMUNICATION STUDY

POST-ENCOUNTER CLINICIAN SURVEY

MAYO CLINIC

Thank you for allowing us to audio-record your encounter with this patient.

Please take a few minutes to complete this brief survey on the clinical encounter that just concluded.

If you have any questions about this study, please contact:
Cara Fernandez, study coordinator: 507-266-1897
Katie James, project coordinator: 507-293-0707

POST-ENCOUNTER CLINICIAN SURVEY

Please answer the following questions about the clinical encounter you just finished with a patient in our study:

1. At what point in the cancer control spectrum is this patient?

☐1 Initial diagnosis
☐2 Early initial treatment
☐3 Mid initial treatment
☐4 Post-treatment/survivorship/remission*
☐5 Recurrence, undergoing treatment
☐6 End-stage disease

* Includes patients undergoing ongoing maintenance (e.g. Tamoxifen)

2. Was a specific decision about this patient's cancer care made during his/her appointment today?

☐1 Yes
☐2 No

3. Please rate how much you agree or disagree with the following statements:

	Strongly disagree	Somewhat disagree	Somewhat agree	Strongly agree
a. I felt that my time with this patient today was well spent.	☐1	☐2	☐3	☐4
b. I established rapport with this patient today.	☐1	☐2	☐3	☐4
c. I was able to obtain an accurate and detailed medical history from this patient.	☐1	☐2	☐3	☐4
d. I think this patient requires a lot of emotional support.	☐1	☐2	☐3	☐4
e. I think that this patient is coping well with his/her cancer treatment and side effects.	☐1	☐2	☐3	☐4
f. Overall, I was satisfied with this consultation today.	☐1	☐2	☐3	☐4

Thank you!
Please return your completed survey to study coordinator,
Cara Fernandez (internal mailing address: Plummer 3 CTSA)

CANCER PATIENT EXPERIENCE AND COMMUNICATION STUDY

POST-ENCOUNTER PATIENT SURVEY

Thank you for participating in our study on communication between cancer patients and their cancer care clinicians.

Please take a few minutes today, immediately following your appointment, to complete this brief survey.

When you are finished, please return the survey to the study coordinator, Cara Fernandez, or the main desk where you checked in for your appointment.

If you have any questions about this study, please contact:

Cara Fernandez, study coordinator: 507-266-1897

Katie James, project coordinator: 507-293-0707

POST-ENCOUNTER PATIENT SURVEY

The term **"clinician"** means a doctor, nurse practitioner, or physician assistant involved in your cancer care. According to our records, you just had an appointment with:

____

Is this information correct? ☐1 Yes ☐2 No → Corrected Name: ____________________

1. <u>Thinking of the visit you just had with your clinician</u>, please select the most appropriate response to each item below:

	Not at all	A little	Somewhat	Quite a bit	Very much
a. How much did your clinician ask to hear exactly what you expected in this visit?	☐1	☐2	☐3	☐4	☐5
b. How much did your clinician try to find out about all your problems and concerns?	☐1	☐2	☐3	☐4	☐5
c. Did you and your clinician decide together which problems and concerns you would talk about today?	☐1	☐2	☐3	☐4	☐5

2. <u>Thinking of the visit you just had with your clinician</u>, please select the most appropriate response to each item below:

	Yes, definitely	Yes, somewhat	No
a. Did your clinician explain things in a way that was easy to understand?	☐1	☐2	☐3
b. Did your clinician listen carefully to you?	☐1	☐2	☐3
c. Did your clinician seem to know the important information about your medical history?	☐1	☐2	☐3
d. Did your clinician show respect for what you had to say?	☐1	☐2	☐3
e. Did your clinician spend enough time with you?	☐1	☐2	☐3

3. Did you have questions or concerns about your care or treatment that you wanted to discuss, but did not?

☐1 Yes →
☐2 No

4. Was a specific decision about your cancer care made during your appointment today?

☐1 Yes → Go to question #5.
☐2 No → Go to question #6.

5. Nine statements related to the decision-making in your consultation are listed below. For each statement, please indicate how much you agree or disagree.

	Completely disagree	Strongly disagree	Somewhat disagree	Somewhat agree	Strongly agree	Completely agree
a. My clinician made it clear that a decision needs to be made.	☐1	☐2	☐3	☐4	☐5	☐6
b. My clinician wanted to know exactly how I wanted to be involved in making the decision.	☐1	☐2	☐3	☐4	☐5	☐6
c. My clinician told me that there are different options for treating my medical conditions.	☐1	☐2	☐3	☐4	☐5	☐6
d. My clinician precisely explained the advantages and disadvantages of the treatment options.	☐1	☐2	☐3	☐4	☐5	☐6
e. My clinician helped me understand all the information.	☐1	☐2	☐3	☐4	☐5	☐6
f. My clinician asked me which treatment I prefer.	☐1	☐2	☐3	☐4	☐5	☐6
g. My clinician and I thoroughly weighed the different treatment options.	☐1	☐2	☐3	☐4	☐5	☐6
h. My clinician and I selected a treatment option together.	☐1	☐2	☐3	☐4	☐5	☐6
i. My clinician and I reached an agreement on how to proceed.	☐1	☐2	☐3	☐4	☐5	☐6

6. Below is a list of topics that some patients discuss with their clinicians. For each topic, please answer the corresponding questions, following the arrows (→) and instructions as you do so.

	Did you discuss this topic?	Did you want to discuss this topic?	Did you discuss this topic as much as you wanted?
**6a. Complementary and Alternative Medicine** (e.g. herbal medicine, acupuncture, homeopathy, meditation)	☐1 Yes →<br>☐2 No →	☐1 Yes →<br>☐2 No → Go to **6b.**	☐1 Yes<br>☐2 No
**6b. Managing symptoms or side effects of treatment** (e.g. pain, nausea, fatigue)	☐1 Yes →<br>☐2 No →	☐1 Yes →<br>☐2 No → Go to **6c.**	☐1 Yes<br>☐2 No
**6c. Emotional or social concerns** (e.g. anxiety, depression, anger, problems with your relationships with others)	☐1 Yes →<br>☐2 No →	☐1 Yes →<br>☐2 No → Go to **#7**	☐1 Yes<br>☐2 No

7. Using any number from 0 to 10, where 0 is the worst clinician possible and 10 is the best clinician possible, what number would you use to rate this clinician?

☐0 ☐1 ☐2 ☐3 ☐4 ☐5 ☐6 ☐7 ☐8 ☐9 ☐10
(worst possible) (best possible)

8. Overall, I was satisfied with my visit to this clinician today.

☐1 Strongly disagree
☐2 Somewhat disagree
☐3 Somewhat agree
☐4 Strongly agree

9. How comfortable were you being audio-recorded today?

☐1 Not at all
☐2 A little bit
☐3 Somewhat
☐4 Quite a bit
☐5 Extremely

10. In 3 months we will mail you a final follow-up survey. In case we need to get in touch with you or remind you about the survey, please provide us with a current phone number where we can reach you.

(__ __ __) __ __ __ - __ __ __ __

Thank you very much for your participation!
Please return your completed survey to the study coordinator, Cara Fernandez, or the main desk where you checked in for your appointment.

Dear <Dr. Last Name>,

We appreciate your continued participation in our study on communication between cancer patients and their clinicians and allowing us to audio-record your encounters with those patients.

There is one final component of this study that will take about 5 minutes of your time.

Clinicians may differ with respect to their comfort level discussing certain topics with their patients, such as complementary and alternative medicine (CAM), psychosocial concerns and end of life issues. With this survey, we hope to learn more about the attitudes and behaviors of clinicians when it comes to discussing these topics with their patients.

Please complete and return the attached survey to our study team in the enclosed pre-addressed, intra-campus mail envelope. Thank you again very much for your participation!

Sincerely,

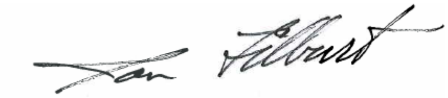

Jon Tilburt, MD and study team

FOLLOW-UP CLINICIAN SURVEY

SECTION A

Complementary and Alternative Medicine (CAM) is "a group of diverse medical and health care systems, practices, and products that are not generally considered part of conventional medicine."

1. In your practice, in what proportion of all cancer patient consultations does a discussion of CAM occur?

_____ % → **If 0%, please skip to question #5**

2. How comfortable are you discussing CAM with your patients?

☐1 Not at all comfortable
☐2 A little comfortable
☐3 Somewhat comfortable
☐4 Very comfortable
☐5 Extremely comfortable

3. In the past 6 months, please estimate the number of patients you have referred to a CAM practitioner or clinic.

______

4. In your experience, at what point in a patient's disease course is a discussion about CAM most likely to occur during the consultation? (Mark only one)

☐1 Initial diagnosis
☐2 Early initial treatment
☐3 Mid initial treatment
☐4 Post-treatment/survivorship/remission
☐5 Recurrence, undergoing treatment
☐6 End-stage disease

5. How beneficial do you think the following services or practices are in promoting patient wellness and/or symptom control in cancer care?

	Not at all beneficial	A little beneficial	Somewhat beneficial	Very beneficial
**HEALTH SERVICES**				
a. Acupuncture	☐1	☐2	☐3	☐4
b. Biofeedback	☐1	☐2	☐3	☐4
c. Chiropractic	☐1	☐2	☐3	☐4
d. Energy healing, Reiki, or healing touch	☐1	☐2	☐3	☐4
e. Hypnotherapy	☐1	☐2	☐3	☐4
f. Massage therapy	☐1	☐2	☐3	☐4
**HEALTH PRACTICES**				
g. Aromatherapy	☐1	☐2	☐3	☐4
h. Guided Imagery	☐1	☐2	☐3	☐4
i. Herbal Medicine	☐1	☐2	☐3	☐4
j. Homeopathy	☐1	☐2	☐3	☐4
k. Meditation	☐1	☐2	☐3	☐4
l. Prayer	☐1	☐2	☐3	☐4
m. Special Diets	☐1	☐2	☐3	☐4
n. Supplements or Mega-vitamins	☐1	☐2	☐3	☐4
o. Tai Chi or Qi Gong	☐1	☐2	☐3	☐4
p. Yoga	☐1	☐2	☐3	☐4

SECTION B

Psychosocial issues discussed with cancer patients may include, but are not limited to, anxiety, depression, anger, and relationship distress.

6. In your practice, in what proportion of all cancer patient consultations does a discussion of psychosocial issues occur?

_____ % → **If 0%, please skip to question #10**

7. How comfortable are you discussing psychosocial issues with your patients?

☐1 Not at all comfortable
☐2 A little comfortable
☐3 Somewhat comfortable
☐4 Very comfortable
☐5 Extremely comfortable

8. In the past 6 months, please estimate the number of patients you have referred to a psychologist or psychiatrist.

_____

9. In your experience, at what point in a patient's disease course is a discussion about psychosocial issues most likely to occur during the consultation? (Mark only one)

☐1 Initial diagnosis
☐2 Early initial treatment
☐3 Mid initial treatment
☐4 Post-treatment/survivorship/remission
☐5 Recurrence, undergoing treatment
☐6 End-stage disease

SECTION C

End-of-life issues discussed with cancer patients may include, but are not limited to, advanced directives, deciding whether to forego additional cancer treatment, and hospice care.

10. In your practice, in what proportion of all cancer patient consultations does a discussion about end-of-life issues occur?

_____ % → **If 0%, please return your completed survey to the study coordinator.**

11. How comfortable are you discussing end-of-life issues with your patients?

☐1 Not at all comfortable
☐2 A little comfortable
☐3 Somewhat comfortable
☐4 Very comfortable
☐5 Extremely comfortable

12. In the past 6 months, please estimate the number of patients you have referred to each of the following for end-of-life issues:

_____ Chaplain _____ Social Worker ______ Palliative Medicine

Thank you again for your help with this study!
Please return your completed survey using the enclosed intra-campus mail envelope.

CANCER PATIENT EXPERIENCE AND COMMUNICATION STUDY
3-MONTH FOLLOW-UP PATIENT SURVEY

MAYO CLINIC

Dear

Thank you for your continued participation in our study on communication between patients with cancer and their clinicians.

About 3 months ago, you completed a short survey about quality of life and communication with your main cancer clinician, who could be a doctor, nurse practitioner, or physician assistant involved in your cancer care. This is a follow-up survey to see how you are doing now.

We hope that your information combined with information from other cancer patients will allow us to improve the quality of care and quality of life for patients in the future.

When you have completed this survey, please place it in the enclosed stamped envelope and mail to the address shown. At this point your participation in our study will be at an end.

Thank you again!

The Mayo Clinic Study Team

Jon C. Tilburt, MD
Kathleen Yost, PhD
Ashok Kumbamu, PhD
Cara Fernandez
Katherine James, MPH

SECTION A – Your Quality of Life

1. Please circle the number (0-10) best reflecting your feelings during the past week, including today. How would you describe:

a. your overall quality of life?

0	1	2	3	4	5	6	7	8	9	10
As bad as it can be										As good as it can be

b. your overall emotional well-being?

0	1	2	3	4	5	6	7	8	9	10
As bad as it can be										As good as it can be

c. your overall physical well-being?

0	1	2	3	4	5	6	7	8	9	10
As bad as it can be										As good as it can be

d. your overall mental (intellectual) well-being?

0	1	2	3	4	5	6	7	8	9	10
As bad as it can be										As good as it can be

e. your level of social activity?

0	1	2	3	4	5	6	7	8	9	10
As bad as it can be										As good as it can be

f. your overall spiritual well-being?

0	1	2	3	4	5	6	7	8	9	10
As bad as it can be										As good as it can be

g. the frequency of your pain?

0	1	2	3	4	5	6	7	8	9	10
No pain										Constant pain

h. the severity of your pain, on the average?

0	1	2	3	4	5	6	7	8	9	10
No pain										Pain as bad as you can imagine

i. your level of fatigue, on the average?

0	1	2	3	4	5	6	7	8	9	10
No fatigue										Constant tiredness

j. your level of support from friends and family?

0	1	2	3	4	5	6	7	8	9	10
No support										Highest level of Support

SECTION A – Your Quality of Life

2. What has been the effect of your cancer diagnosis and treatment on <u>your</u> overall quality of life? (Please circle the appropriate number)

0	1	2	3	4	5	6	7	8	9	10
Totally ruined quality of life										No effect on quality of life

Comments:____

3. What has been the effect of your cancer diagnosis and treatment on <u>your family's</u> quality of life? (Please circle the appropriate number)

0	1	2	3	4	5	6	7	8	9	10
Totally ruined quality of life										No effect on quality of life

Comments:____

4. Compared to <u>3 months ago</u>, how would you describe your overall quality of life <u>now</u>? (Check ONE box below)

☐1 A lot worse quality of life

☐2 A little worse quality of life

☐3 About the same quality of life

☐4 A little better quality of life

☐5 A lot better quality of life

SECTION B – Making Decisions about Cancer Care

For the next 2 questions, the term **"clinician"** means a doctor, nurse practitioner, or physician assistant involved in your cancer care

5. Which of these five statements best describes the role you would prefer to have in decisions about your cancer care? (Please circle the most appropriate letter)

A	B	C	D	E
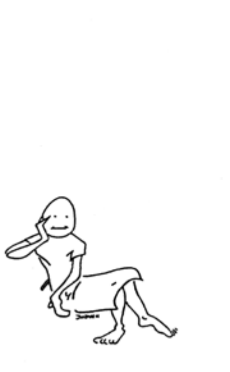	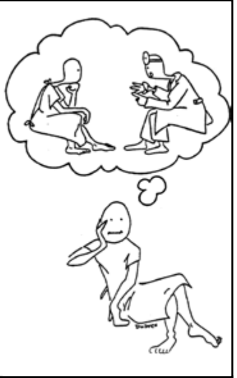	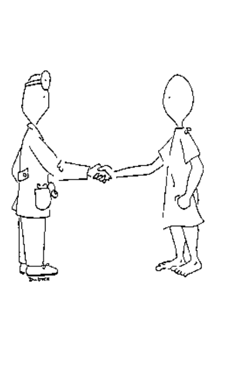	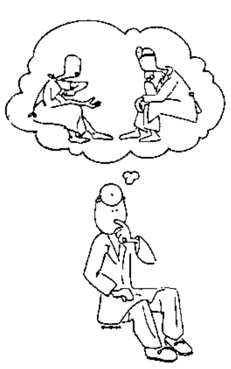	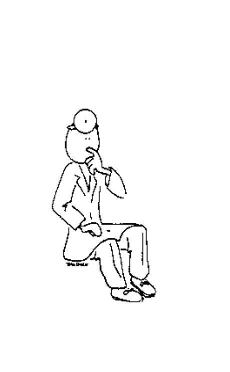
I prefer to make decisions about my health care.	I prefer to make decisions about my health care after seriously considering my cancer clinician's opinion.	I prefer that my cancer clinician and I share responsibility for making decisions about my health care.	I prefer that my cancer clinician make decisions about my health care, but seriously considers my opinion.	I prefer to leave decisions about my health care to my cancer clinician.

6. Which of these five statements best describes the role you actually have had in decisions about your cancer care? (Please circle the most appropriate box)

A	B	C	D	E
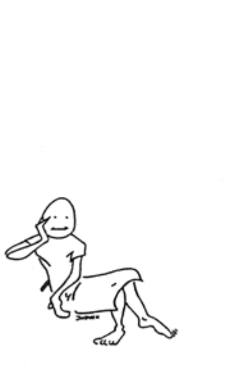	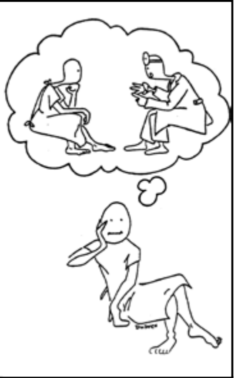	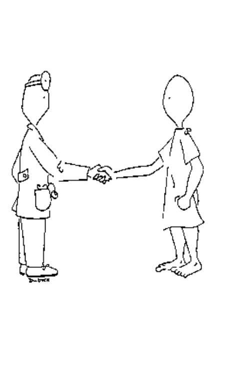	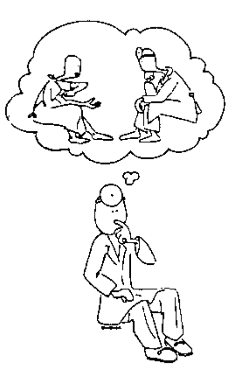	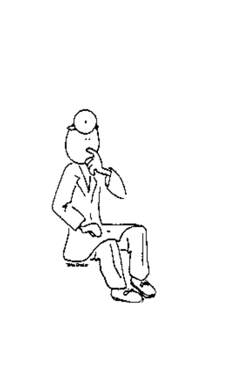
I made all the decisions about my health care.	I made all the decisions about my health care after seriously considering my cancer clinician's opinion.	My cancer clinician and I shared the responsibility for deciding what was best for me.	My cancer clinician made decisions about my cancer after seriously considers my opinion.	I left all the decisions about my cancer to my cancer clinician.

SECTION C - Complementary and Alternative Medicine

The following questions are about some health services that typically require you to see a practitioner. They are listed in alphabetical order. A glossary of terms can be found on page 10-11.

**Type of Health Service**	**When did you see a provider or practitioner for this service? (Mark one)**	**Did you see a provider or practitioner for this service in the last 3 months?**
Acupuncture	☐ Never → **go to next type of service**<br>☐ Only before cancer diagnosis<br>☐ Only after cancer diagnosis<br>☐ Before & after cancer diagnosis	☐ Yes<br>☐ No
Biofeedback	☐ Never → **go to next type of service**<br>☐ Only before cancer diagnosis<br>☐ Only after cancer diagnosis<br>☐ Before & after cancer diagnosis	☐ Yes<br>☐ No
Chiropractic care	☐ Never → **go to next type of service**<br>☐ Only before cancer diagnosis<br>☐ Only after cancer diagnosis<br>☐ Before & after cancer diagnosis	☐ Yes<br>☐ No
Energy Healing, Reiki or Healing Touch	☐ Never → **go to next type of service**<br>☐ Only before cancer diagnosis<br>☐ Only after cancer diagnosis<br>☐ Before & after cancer diagnosis	☐ Yes<br>☐ No
Hypnotherapy	☐ Never → **go to next type of service**<br>☐ Only before cancer diagnosis<br>☐ Only after cancer diagnosis<br>☐ Before & after cancer diagnosis	☐ Yes<br>☐ No
Massage therapy	☐ Never → **go to next type of service**<br>☐ Only before cancer diagnosis<br>☐ Only after cancer diagnosis<br>☐ Before & after cancer diagnosis	☐ Yes<br>☐ No
Other practitioner (e.g. traditional healer)	☐ Never → **go page 6**<br>☐ Only before cancer diagnosis<br>☐ Only after cancer diagnosis<br>☐ Before & after cancer diagnosis	☐ Yes<br>☐ No

Since being diagnosed with cancer, did you use this service for any of these reasons? (Mark all that apply)	Since being diagnosed with cancer, did you let your main cancer clinician know about your use of this service?	What role did your main cancer clinician have in your use of this type of service? (Mark one)
☐ To fight the cancer<br>☐ To manage side effects of cancer treatment<br>☐ To improve well-being<br>☐ Other: ____________	☐ Yes<br>☐ No	☐ No role<br>☐ He/she had no opinion about my use of this service<br>☐ He/she suggested I use this service<br>☐ He/she cautioned against my use of this service
☐ To fight the cancer<br>☐ To manage side effects of cancer treatment<br>☐ To improve well-being<br>☐ Other: ____________	☐ Yes<br>☐ No	☐ No role<br>☐ He/she had no opinion about my use of this service<br>☐ He/she suggested I use this service<br>☐ He/she cautioned against my use of this service
☐ To fight the cancer<br>☐ To manage side effects of cancer treatment<br>☐ To improve well-being<br>☐ Other: ____________	☐ Yes<br>☐ No	☐ No role<br>☐ He/she had no opinion about my use of this service<br>☐ He/she suggested I use this service<br>☐ He/she cautioned against my use of this service
☐ To fight the cancer<br>☐ To manage side effects of cancer treatment<br>☐ To improve well-being<br>☐ Other: ____________	☐ Yes<br>☐ No	☐ No role<br>☐ He/she had no opinion about my use of this service<br>☐ He/she suggested I use this service<br>☐ He/she cautioned against my use of this service
☐ To fight the cancer<br>☐ To manage side effects of cancer treatment<br>☐ To improve well-being<br>☐ Other: ____________	☐ Yes<br>☐ No	☐ No role<br>☐ He/she had no opinion about my use of this service<br>☐ He/she suggested I use this service<br>☐ He/she cautioned against my use of this service
☐ To fight the cancer<br>☐ To manage side effects of cancer treatment<br>☐ To improve well-being<br>☐ Other: ____________	☐ Yes<br>☐ No	☐ No role<br>☐ He/she had no opinion about my use of this service<br>☐ He/she suggested I use this service<br>☐ He/she cautioned against my use of this service
☐ To fight the cancer<br>☐ To manage side effects of cancer treatment<br>☐ To improve well-being<br>☐ Other: ____________	☐ Yes<br>☐ No	☐ No role<br>☐ He/she had no opinion about my use of this service<br>☐ He/she suggested I use this service<br>☐ He/she cautioned against my use of this service

SECTION C - Complementary and Alternative Medicine

The following questions are about some health practices that typically require you to see a practitioner. They are listed in alphabetical order. A glossary of terms can be found on page 10-11.

**Type of Health Practice**	**When did you use/do this practice? (Mark one)**	**Did you use/do this practice in the last 3 months?**
Aromatherapy	☐ Never → **go to next type of practice**<br>☐ Only before cancer diagnosis<br>☐ Only after cancer diagnosis<br>☐ Before & after cancer diagnosis	☐ Yes<br>☐ No
Guided Imagery	☐ Never → **go to next type of practice**<br>☐ Only before cancer diagnosis<br>☐ Only after cancer diagnosis<br>☐ Before & after cancer diagnosis	☐ Yes<br>☐ No
Herbal Medicine	☐ Never → **go to next type of practice**<br>☐ Only before cancer diagnosis<br>☐ Only after cancer diagnosis<br>☐ Before & after cancer diagnosis	☐ Yes<br>☐ No
Homeopathy	☐ Never → **go to next type of practice**<br>☐ Only before cancer diagnosis<br>☐ Only after cancer diagnosis<br>☐ Before & after cancer diagnosis	☐ Yes<br>☐ No
Meditation	☐ Never → **go to next type of practice**<br>☐ Only before cancer diagnosis<br>☐ Only after cancer diagnosis<br>☐ Before & after cancer diagnosis	☐ Yes<br>☐ No
Prayer	☐ Never → **go to page 8**<br>☐ Only before cancer diagnosis<br>☐ Only after cancer diagnosis<br>☐ Before & after cancer diagnosis	☐ Yes<br>☐ No

**<u>Since being diagnosed</u> with cancer, did you use this practice for any of these reasons? (Mark <u>all</u> that apply)**	**<u>Since being diagnosed</u> with cancer, did you let your main cancer clinician know about your use of this practice?**	**What role did your main cancer clinician have in your use of this type of practice? (Mark <u>one</u>)**
☐ To fight the cancer<br>☐ To manage side effects of cancer treatment<br>☐ To improve well-being<br>☐ Other: ___________	☐ Yes<br>☐ No	☐ No role<br>☐ He/she had no opinion about my use of this practice<br>☐ He/she suggested I use this practice<br>☐ He/she cautioned against my use of this service
☐ To fight the cancer<br>☐ To manage side effects of cancer treatment<br>☐ To improve well-being<br>☐ Other: ___________	☐ Yes<br>☐ No	☐ No role<br>☐ He/she had no opinion about my use of this practice<br>☐ He/she suggested I use this practice<br>☐ He/she cautioned against my use of this practice
☐ To fight the cancer<br>☐ To manage side effects of cancer treatment<br>☐ To improve well-being<br>☐ Other: ___________	☐ Yes<br>☐ No	☐ No role<br>☐ He/she had no opinion about my use of this practice<br>☐ He/she suggested I use this practice<br>☐ He/she cautioned against my use of this service
☐ To fight the cancer<br>☐ To manage side effects of cancer treatment<br>☐ To improve well-being<br>☐ Other: ___________	☐ Yes<br>☐ No	☐ No role<br>☐ He/she had no opinion about my use of this practice<br>☐ He/she suggested I use this practice<br>☐ He/she cautioned against my use of this service
☐ To fight the cancer<br>☐ To manage side effects of cancer treatment<br>☐ To improve well-being<br>☐ Other: ___________	☐ Yes<br>☐ No	☐ No role<br>☐ He/she had no opinion about my use of this practice<br>☐ He/she suggested I use this practice<br>☐ He/she cautioned against my use of this service
☐ To fight the cancer<br>☐ To manage side effects of cancer treatment<br>☐ To improve well-being<br>☐ Other: ___________	☐ Yes<br>☐ No	☐ No role<br>☐ He/she had no opinion about my use of this practice<br>☐ He/she suggested I use this practice<br>☐ He/she cautioned against my use of this service

SECTION C - Complementary and Alternative Medicine

The following questions are about some health practices that typically require you to see a practitioner. They are listed in alphabetical order. A glossary of terms be found on page 10-11.

Type of Health Practice	When did you use/do this practice? (Mark one)	Did you use/do this practice in the last 3 months?
Special Diets	☐ Never → **go to next type of practice**<br>☐ Only before cancer diagnosis<br>☐ Only after cancer diagnosis<br>☐ Before & after cancer diagnosis	☐ Yes<br>☐ No
Supplements or Mega-vitamins	☐ Never → **go to next type of practice**<br>☐ Only before cancer diagnosis<br>☐ Only after cancer diagnosis<br>☐ Before & after cancer diagnosis	☐ Yes<br>☐ No
Tai Chi or Qi Gong	☐ Never → **go to next type of practice**<br>☐ Only before cancer diagnosis<br>☐ Only after cancer diagnosis<br>☐ Before & after cancer diagnosis	☐ Yes<br>☐ No
Yoga	☐ Never → **go to next type of practice**<br>☐ Only before cancer diagnosis<br>☐ Only after cancer diagnosis<br>☐ Before & after cancer diagnosis	☐ Yes<br>☐ No
Other practice (e.g. home remedy)	☐ Never<br>☐ Only before cancer diagnosis<br>☐ Only after cancer diagnosis<br>☐ Before & after cancer diagnosis	☐ Yes<br>☐ No

SECTION C - Complementary and Alternative Medicine

**<u>Since being diagnosed</u> with cancer, did you use this practice for any of these reasons? (Mark <u>all</u> that apply)**	**<u>Since being diagnosed</u> with cancer, did you let your main cancer clinician know about your use of this practice?**	**What role did your main cancer clinician have in your use of this type of practice? (Mark <u>one</u>)**
☐ To fight the cancer<br>☐ To manage side effects of cancer treatment<br>☐ To improve well-being<br>☐ Other: ___________	☐ Yes<br>☐ No	☐ No role<br>☐ He/she had no opinion about my use of this practice<br>☐ He/she suggested I use this practice<br>☐ He/she cautioned against my use of this practice
☐ To fight the cancer<br>☐ To manage side effects of cancer treatment<br>☐ To improve well-being<br>☐ Other: ___________	☐ Yes<br>☐ No	☐ No role<br>☐ He/she had no opinion about my use of this practice<br>☐ He/she suggested I use this practice<br>☐ He/she cautioned against my use of this practice
☐ To fight the cancer<br>☐ To manage side effects of cancer treatment<br>☐ To improve well-being<br>☐ Other: ___________	☐ Yes<br>☐ No	☐ No role<br>☐ He/she had no opinion about my use of this practice<br>☐ He/she suggested I use this practice<br>☐ He/she cautioned against my use of this practice
☐ To fight the cancer<br>☐ To manage side effects of cancer treatment<br>☐ To improve well-being<br>☐ Other: ___________	☐ Yes<br>☐ No	☐ No role<br>☐ He/she had no opinion about my use of this practice<br>☐ He/she suggested I use this practice<br>☐ He/she cautioned against my use of this practice
☐ To fight the cancer<br>☐ To manage side effects of cancer treatment<br>☐ To improve well-being<br>☐ Other: ___________	☐ Yes<br>☐ No	☐ No role<br>☐ He/she had no opinion about my use of this practice<br>☐ He/she suggested I use this practice<br>☐ He/she cautioned against my use of this practice

Thank you! You have completed the survey.

Glossary of Complementary and Alternative Medicine Terminology

Acupuncture – one of the oldest healing practices in the world, acupuncture is a family of procedures in which specific points on the body (i.e. "acupoints") are stimulated using a variety of techniques (e.g. penetrating the skin with needles, electrical stimulation) most commonly to treat pain.

Aromatherapy – a therapy in which the scent of oils from flowers, herbs, and trees is inhaled to promote health and well-being.

Biofeedback – learned techniques used by a patient to consciously regulate bodily functions such as breathing, heart rate, and blood pressure. Biofeedback is commonly used to reduce stress, eliminate headaches, relieve pain, and recondition injured muscles.

Chiropractic care – a therapy which involves adjusting the spinal column, joints and improving alignment or positioning of the vertebrae. It is primarily used to treat back problems, headaches, nerve inflammation, muscle spasms, and other injuries/traumas.

Energy Healing – a type of healing aimed at restoring and balancing the natural life force energy within the body by the channeling of healing energy through the hands of a practitioner into the patient's body.

Guided Imagery – involves a series of relaxation techniques followed by the visualization of detailed images. A practitioner describes a scene or mental image for the patient to focus on, such as an image of the patient's body free of the specific problem or condition. Guided imagery has been used to help with alleviate stress, anxiety, high blood pressure, headaches, and to control pain or side effects of cancer therapy or surgery.

Healing Touch – this practice, derived from the ancient technique of "laying-on of hands," is based on the premise that the healing force of the therapist can impact the patient's recovery and that healing is promoted when there is a balance of the body's energies.

Herbal Medicine – also referred to as botanical medicine, is the use of a plant's seeds, berries, roots, leaves, bark, or flowers for purposes of improving health, preventing disease, and treating illness. Examples of widely used herbal medicines include echinacea, St. John's wort, ginkgo, ginseng, and garlic.

Homeopathy – a system of medical practices based on the theory that substances that make you ill can also cure you (e.g. giving a homeopathic dose of onions to alleviate symptoms of a common cold, or coffee to a person suffering from insomnia). Homeopathic remedies are derived from many natural sources such as plants, metals, and minerals and are given in very small doses.

Glossary of Complementary and Alternative Medicine Terminology

Hypnotherapy – also known as hypnosis, involves the power of suggestion by a trained hypnotist during a deep state of relaxation. This therapy, similar to meditation and mental imagery, is used to effect positive changes and treat numerous health conditions including ulcers, chronic pain, respiratory ailments, stress, and headaches.

Massage Therapy – a type of therapy in which a practitioner manipulates muscle and connective tissue to enhance function of those tissues and aid in relaxation, lower blood pressure, and improve circulation.

Meditation – a group of techniques, many of which started in Eastern religious or spiritual traditions, that involve contemplation, reflection, or directing one's attention towards particular thoughts or concepts. Meditation is often used to induce mental tranquility and physical relaxation.

Prayer – an active process of appealing to a higher spiritual power which is done either individually or in a group on behalf of oneself or others.

Qi Gong – an ancient Chinese discipline that uses gentle, repetitive physical movements, mental focus, and deep breathing directed toward specific parts of the body.

Reiki – a type of energy medicine in which a practitioner places his or her hands on or near the patient with the intent to transmit *ki*, believed to be life-force energy. Reiki originated in Japan.

Special Diets – diet-based therapies aimed at enhancing health, examples include Atkins, Pritikin, Ornish, and Zone.

Supplements or Mega-vitamins – supplements are substances or products added to a person's diet to ensure they receive the nutrients they need. This can include vitamins, minerals, protein, or fat. Mega-vitamin therapy refers to taking vitamins in doses that exceed their recommended daily allowance (RDA).

Tai Chi – a Chinese mind-body practice that involves moving one's body slowly and gently, while breathing deeply and meditating. It is thought to improve the flow throughout the body of a vital energy called "qi."

Yoga – an ancient system of practices used to balance the mind and body through exercise, meditation, and control of breathing and emotions. When most people hear the term yoga, they usually think of *Hatha* or *Kundalini* yoga, which involves the adoption of various bodily postures or poses. It is commonly used for relaxation or to improve flexibility and balance.

Thank you again for your participation in this study! Please do not hesitate to contact our research team if you have any questions.

(507) 266-1897

fernandez.cara@mayo.edu
